# Home blood pressure monitors owned by participants in a large decentralised clinical trial in hypertension: the Treatment In Morning versus Evening (TIME) study

**DOI:** 10.1038/s41371-021-00496-6

**Published:** 2021-02-15

**Authors:** Thineskrishna Anbarasan, Amy Rogers, David A. Rorie, J. W. Kerr Grieve, Thomas M. MacDonald, Isla S. Mackenzie

**Affiliations:** 1 Medical Student, University of Dundee, Dundee, UK; 2 Clinical Research Fellow, University of Dundee, Dundee, UK; 3 Senior Software Developer, University of Dundee, Dundee, UK; 4 Professor of Clinical Pharmacology and Pharmacoepidemiology, University of Dundee, Dundee, UK; 5 Professor of Cardiovascular Medicine, MEMO Research, Division of Molecular and Clinical Medicine, University of Dundee, Dundee, UK

**Keywords:** Hypertension, Clinical trials, Diagnosis

## Abstract

Various home blood pressure monitors (HBPMs) are available to the public for purchase but only some are validated against standardised protocols. This study aimed to assess whether HBPMs owned by participants taking part in a clinical trial were validated models. The TIME study is a decentralised randomised trial investigating the effect of antihypertensive medication dosing time on cardiovascular outcomes in adults with hypertension. No HBPMs were provided to participants in this trial but patients were asked to report if they already owned one. We identified the model of HBPM reported by participants, then cross-referenced this against lists of validated HBPMs produced by dabl Educational Trust and the British and Irish Hypertension Society (BIHS). Of 21,104 participants, 10,464 (49.6%) reported their model of HBPM. 7464 (71.3%) of these participants owned a monitor that could be identified from the participants’ entry. Of these, 6066 (81.3%) participants owned a monitor listed as validated by either dabl (*n* = 5903) or BIHS (*n* = 5491). Some were listed as validated by both. 1398 (18.7%) participants owned an identifiable HBPM that lacked clear evidence of validation. 6963 (93.3%) participants owned an upper arm HBPM and 501 (6.7%) owned a wrist HBPM. Validated HBPMs had a higher median online retail price of £45.00 compared to £20.00 for HBPMs lacking clear evidence of validation. A significant number of participants own HBPMs lacking evidence of validation.

## Introduction

High blood pressure or hypertension is an important risk factor for cardiovascular disease [1, 2] and the greatest preventable risk factor for disability and premature death worldwide [3]. It is well established that adequately controlled hypertension reduces the risk of cardiovascular disease and all-cause mortality [4]. While non-invasive blood pressure monitoring using auscultatory sphygmomanometers and automated oscillatory blood pressure monitors can only provide an approximation of intra-arterial blood pressure, such methods are the mainstay of clinical diagnosis and management. Increasingly, hypertension guidelines are recognising the importance of out-of-clinic blood pressure measurements, particularly with home blood pressure monitors (HBPMs) as a key element in the diagnosis and management of hypertension [5–8].

The use of HBPMs offers several potential advantages over clinic blood pressure measurements including the provision of multiple readings over an extended period of time, convenience for patients, avoidance of white coat effect and increased awareness and interest in self-management of hypertension amongst patients [9, 10]. Home blood pressure measurements have been found to be more reproducible [11] and a better predictor of cardiovascular mortality in comparison to clinic measurements [12, 13]. Use of HBPMs by patients to self-monitor blood pressure, with appropriate support (including education and systematic titration of medication), has been associated with improved control compared to patients managed with only clinic blood pressure monitoring [14, 15].

For the advantages of hypertension self-monitoring to be realised, it is imperative that the HBPMs used provide accurate blood pressure measurements. Various HBPMs are available for purchase but only some are validated against independent standards; this may affect the accuracy of blood pressure measurements using these monitors [16]. Often, trials investigating the efficacy of interventions using HBPMs provide participants with validated monitors therefore it is unclear if the findings of such trials can be generalised to usual practice where patients may be expected to purchase their own monitor. The recent ACCU-RATE study reported that unvalidated monitors owned by patients were less likely to pass standardised static pressure testing than validated (64% pass rate vs 96%) [17].

In this study, we aim to describe the validation status of HBPMs already owned by participants in the Treatment In Morning versus Evening (TIME) study. Additionally, we aim to determine if validation status of HBPM owned is associated with the price of the HBPM and socioeconomic status of participants.

## Methods

### Study participants

The TIME study is a prospective, randomised, open-label, blinded end-point (PROBE) design trial investigating the effect of antihypertensive medication dosing time on cardiovascular outcomes in 21,104 adults with hypertension. The TIME study is an example of a remote decentralised clinical trial with a single central site and remote participation. Patients who were aged over 18 years, prescribed at least one antihypertensive medication to be taken once daily and had a valid e-mail address were eligible to enrol in the TIME study via a secure web-portal (https://www.timestudy.co.uk). Recruitment for the TIME study was via advertising to eligible patients across the UK from primary care, secondary care and from databases of individuals who have consented to be approached for participation in research studies. Potential participants were invited to complete an online questionnaire which required confirmation of study suitability based on the inclusion and exclusion criteria as detailed in the study protocol [18]. Following confirmation of eligibility, participants could complete the consent and registration process online prior to randomisation. The TIME study utilises an information technology-based methodology to monitor patient outcomes.

The TIME study is a registered clinical trial (EudraCT 2011-001968-21, ISRCTN18157641) with ethical approval (East of Scotland Research Ethics Service 11/AL/0309).

### Data collection

All TIME study participants were asked if they owned an HBPM and if they would be willing to provide measurements for the study. A drop-down menu with commonly used HBPM models was provided for participants to select from. In this menu, an option labelled “Other Not Specified” was available for participants owning an HBPM model not featured in the drop-down menu. Patients who selected the option “Other Not Specified” had access to a free-text field to input the model of their HBPM. All free-text entries were subsequently interpreted and matched against commercially available models.

**Fig. 1:**
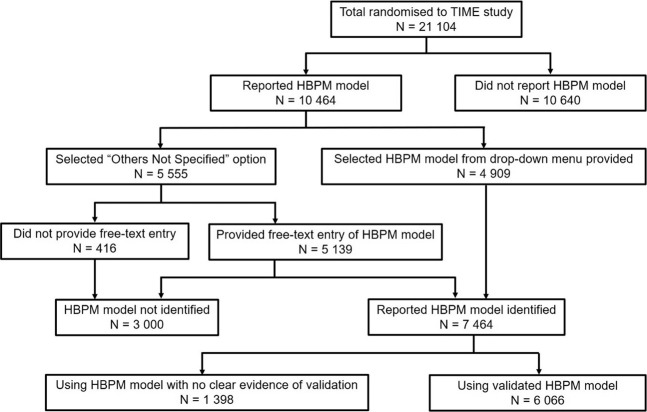
Flowchart showing derivation of study population used in this analysis. Number of individual participants (N), Home blood pressure monitor (HBPM).

Due to the unstructured nature of free-text entries, several entries from participants could not be deciphered to accurately determine the HBPM model in use. These entries were therefore not included for subsequent analysis. A study schematic with numbers of participant entries matched to a HBPM model is shown in Fig. 1.

**Fig. 2:**
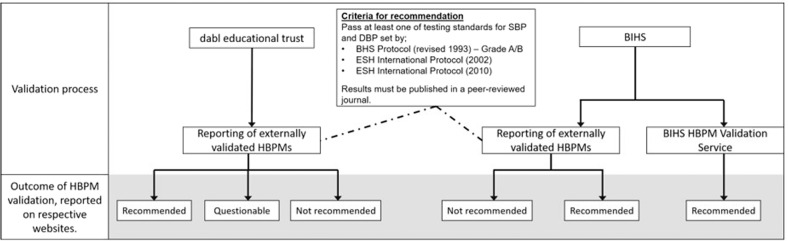
A flow diagram explaining the reporting of HBPM validation status by dabl Education Trust and the BIHS. Diastolic blood pressure (DBP), Systolic blood pressure (SBP), British Hypertension Society (BHS), European Society of Hypertension (ESH), Home blood pressure monitor (HBPM).

To determine the validation status of HBPMs in use, identified models were cross-referenced against lists of validated HBPMs produced by dabl Educational Trust [19] and the British and Irish Hypertension Society (BIHS) [20]. Both the dabl Educational Trust and BIHS have differing reporting practices, as shown in Fig. 2, but adhere to recommending HBPMs which pass at least one of the testing standards established by the British Hypertension Society protocol [21], European Society of Hypertension (ESH) International protocol (2002) [22] or ESH International protocol (2010) [23]. In this study we classified HBPMs as having clear evidence of validation if it was recommended as validated by either BIHS or the dabl Educational Trust. HBPMs which were reported as having questionable evidence or not reported by either organisation, were classified as having no clear evidence of validation.

To determine the influence of participants’ socioeconomic status on choice of HBPM, we derived socioeconomic deprivation scores, based on participants’ residential postcode. Participants in the TIME study were recruited from different parts of the UK (i.e. England, Wales, Scotland and Northern Ireland). Postcodes were used to assign each participants’ individual index of multiple deprivation (IMD) decile scores from respective national statistics websites:

- Scotland (https://www.gov.scot/Topics/Statistics/SIMD);
- England (https://imd-by-postcode.opendatacommunities.org);
- Wales (https://gov.wales/statistics-and-research/welsh-index-multiple-deprivation/?lang=en);
- Northern Ireland (https://www.nisra.gov.uk/statistics/deprivation/northern-ireland-multiple-deprivation-measure-2017-nimdm2017).

The socioeconomic deprivation decile scores from the different constituent countries of UK, were aggregated as a combined IMD decile score. Subsequently, participants were classified into either being in the less deprived (IMD decile 6–10) or more deprived (IMD decile 1–5) socioeconomic groups for analysis. Country-specific variations in IMD decile scores, were adjusted for with the inclusion of the country of residence as a separate variable in multivariate analysis.

A web search was performed to determine the online retail price of identified HBPMs. The lowest online retail price for a HBPM was searched (April 2018) on trusted web sources (e.g. www.bloodpressureuk.org) and common online retailers (e.g. www.amazon.co.uk). If no price could be found, a wider web search was performed to identify a listed price. The lowest online retail price for new (first-hand) HBPMs were included for analysis. HBPMs with no available price were excluded from the cost analysis.

### Statistical analysis

Data was summarised as number of patients and percentage for categorical variables. Prices of HBPMs are reported as medians with Kruskal–Wallis test and Dunn’s post hoc test performed to determine significant differences. A logistic regression model was constructed to determine participant-level and HBPM related factors which are associated with the reported HBPM having clear evidence of validation. All analysis was performed on RStudio version 3.5.1 (RStudio, Inc, Massachusetts, USA). A *p* value < 0.05 was considered to be statistically significant.

## Results

### Description of HBPMs owned

A total of 21,104 participants were randomised to the TIME study. Participants recruited to the TIME study were from England (*n* = 18,532; 87.8%), Scotland (*n* = 1816; 8.6%), Wales (*n* = 750; 3.6%) and Northern Ireland (*n* = 6; 0.03%). A total of 11,434 (54.2%) participants reported owning an HBPM and 10,464 (49.6%) reported the model of HBPM owned.

From the participants who reported their model of HBPM, 4909 (46.9%) owned a model which was available for selection from the provided drop-down menu in the TIME study web interface. The remaining 5555 (53.1%) participants had selected the option “Other Not Specified” gaining access to input their model of HBPM owned in the free-text entry field. Of these, 3000 entries either could not be interpreted or were left unfilled and the underlying HBPM model therefore not identified. The exact model of HBPM owned was determined for 7464 (71.3%) participants. A total of 261 different HBPM models were reported of which 187 were upper arm models and 74 were wrist models. Most participants, 6963 (93.3%), owned an upper arm HBPM with 501 (6.7%) participants owning a wrist HBPM. Only one participant reported owning a manual HBPM with the remainder owning an automatic HBPM. Five common models comprised just over half (53%) of identifiable HBPMs, and 49% were built by one dominant manufacturer. The prices of 175 (67.0%) HBPM models owned by 7077 participants were identified. The median price of an HBPM owned by participants in the TIME study was £45.00. The median price of wrist HBPMs was significantly higher than upper arm HBPMs (£120.00 vs £45.00; *p* < 0.001).

Amongst the 7464 participants whose HBPM models were identified, the postal codes were available for 7395 (99.1%) participants. Of these, 1799 (24.3%) participants resided in more deprived socioeconomic regions (IMD deciles 1–5).

### Validation of HBPMs owned

From a total of 7464 participants, whose HBPM models were identified, 6066 (81.3%) participants owned a model which had evidence of validation. Of these, the HBPMs owned by 5903 (79.1%) and 5491 (73.6%) participants had evidence of validation reported in the dabl Educational Trust and BIHS databases respectively. The median price of validated HBPMs was significantly higher than that of HBPMs lacking clear evidence of validation (£45.00 vs £20.00, *p* < 0.001). The median expenditure on HBPMs was £45.00 amongst participants from both less deprived regions (IMD deciles 6–10) and more deprived regions (IMD deciles 1–5).

A multivariate logistic regression model based on participants’ socioeconomic deprivation group (less deprived vs more deprived), type of HBPM (upper arm vs wrist), median price of HBPM, degree of participant engagement with self-reporting of BP readings (measured by whether a participant had submitted at least one set of systolic and diastolic blood pressure readings) and country of residence at enrolment was constructed (Table 1). At least one set of systolic and diastolic blood pressure readings were available from 5389 (72.2%) participants of which 4401 owned a validated monitor. Participants who purchased upper arm HBPMs (adjusted OR, 5.08 (95% CI, 3.83–6.75)) or HBPMs costing greater than the median online price of £45.00 (adjusted OR, 14.4 (95% CI, 11.2–18.7)) or whose socioeconomic status could not be derived (adjusted OR, 2.62 (95% CI, 1.26–5.96)) were significantly more likely to own a model having clear evidence of validation. Participants who owned HBPMs with no available online retail price (adjusted OR, 0.08 (95% CI, 0.06–0.11)) or resided in Scotland (adjusted OR, 0.65 (95% CI, 0.50–0.83)) compared to England at the time of study enrolment were significantly less likely to own a model with evidence of validation.

**Table 1:** HBPM and participant characteristics associated with the likelihood of HBPM model owned having clear evidence of validation.

	HBPM with clear evidence of validation?		Univariate analysis			Adjusted analysis		
	Yes (%)	No (%)	OR	95% CI	*P* value	OR	95% CI	*P* value
Subgroup HBPM characteristics								
Type of monitor								
Wrist	281 (4.6)	220 (15.4)	Reference					
Upper arm	5785 (95.4)	1178 (84.3)	3.85	(3.19–4.64)	<0.001	5.08	3.83–6.75	<0.001
Price of HBPM								
≤ than median (£45.00)	3300 (54.4)	1004 (71.8)	Reference					
> than median (£45.00)	2700 (44.5)	73 (5.2)	11.3	(8.89–14.5)	<0.001	14.4	11.2–18.7	<0.001
Not known	66 (1.1)	321 (23.0)	0.06	(0.05–0.08)	<0.001	0.08	0.06–0.11	<0.001
Participant characteristics								
At least 1 BP reading available								
No	1665 (22.3)	410 (5.5)	Reference					
Yes	4401 (59.0)	988 (13.2)	1.1	(0.96–1.25)	0.157	0.95	0.82–1.10	0.512
Socioeconomic status								
More deprived^a^	1439 (23.7)	360 (25.7)	Reference					
Less deprived^b^	4569 (75.3)	1027 (73.5)	1.11	(0.97–1.27)	0.117	1.13	0.97–1.31	0.118
Not known	58 (1.0)	11 (0.8)	1.31	(0.71–2.68)	0.407	2.62	1.26–5.96	0.015
Country of residence at enrolment								
England	5486 (73.5)	1238 (16.6)	Reference					
Scotland	398 (5.3)	117 (1.6)	0.77	(0.62–0.96)	0.016	0.65	0.50–0.83	0.001
Wales	182 (2.4)	43 (0.6)	0.96	(0.69–1.36)	0.79	1.04	0.71–1.55	0.845
Northern Ireland	0	0						

^a^More deprived (IMD decile 1–5).

^b^Less deprived (IMD decile 6–10).

## Discussion

This study offers a pragmatic insight into the type of HBPMs used by patients with hypertension in the UK. A large variety of HBPMs, most commonly manufactured by Omron, are used by the TIME study participants. Upper arm HBPMs are more commonly used in comparison to wrist HBPMs with the latter observed to have higher median price. Wrist HBPMs offer an advantage of smaller size and greater ease of use compared to upper arm HBPMs [24, 25]. The more recent introduction of wrist HBPMs to the market, higher price points, and lack of endorsement in guidelines may also explain why wrist HBPMs were less prevalent than upper arm models.

Most participants who reported their HBPM model, owned a model validated by the dabl Education Trust or BIHS. However, a significant number (*n* = 1398; 18.7%) owned a HBPM lacking clear evidence of validation. Information on the validation status of a HBPM may be omitted by manufacturers on their packaging or product information leaflets, or, where present, may be obscured by excessive branding. Phrases such as “clinically proven” or “clinically tested” do not equate to validation against independent standards and may entice the uninformed patient into inadvertently purchasing a HBPM without validation [26]. Additionally, statements such as “FDA cleared” are included under the description of some unvalidated HBPMs. The FDA approval certification for HBPMs is to assure device safety in operation rather than affirming the clinical accuracy of HBPMs [27].

HBPMs lacking clear evidence of validation had a lower median price than validated HBPMs in our study. Some patients may have been inclined to purchase the former, unaware that guidelines recommend the use of validated monitors. A recent study investigating the validation status of HBPMs in use by patients in Turkey reported that only 36% had evidence of validation reported by the dabl Educational Trust or BIHS websites [28] however the English ACCU-RATE study found 69% [17]. The proportion of validated monitors in use was also higher in our cohort of patients. This may reflect national differences in regulations for the import and sale of HBPMs. With increasing numbers of medical devices being bought online, it becomes easier for manufacturers to bypass local regulations to market unvalidated monitors to patients [29]. A study investigating the validation of HBPMs available for purchase through the internet found that only 66 out of 124 websites offered at least one validated HBPM and of these, only 6 sites had information on the exact validation protocol passed by the HBPM on sale [26].

Wrist HBPMs were significantly associated with a reduced likelihood of having a clear evidence of validation. Wrist HBPMs require strict adherence to posture with the monitor needing to be placed at heart level to achieve accurate measurements. Even validated wrist HBPMs with position sensors have been observed to overestimate BP measurements in patients self-monitoring BP [30]. Given this reputation of wrist HBPMs for inaccuracy [31], it is possible that several manufacturers may have chosen not to submit their monitors for validation procedure prior to marketing.

Although validated HBPMs were of significantly higher price than HBPMs lacking clear evidence of validation, the ownership of the former was not associated with the participants’ socioeconomic status (derived from residential postcode). In fact, the median prices of HBPMs owned by participants from different socioeconomic groups were largely similar. This suggests that spending on HBPMs by participants in our study may be independent of socioeconomic status. For this group of patients, additional information on the importance of choosing *validated* HBPMs may prove more effective than financial incentives in guiding purchase decisions.

Several participants (*n* = 809) had entered the vendor’s name (e.g. “Lloyds” or “Boots”) or model of BP cuff used or indicated that they did not know/could not find the model of their HBPM. Several others had only indicated the brand name (e.g. Omron or Braun) with no indication of the specific model. This uncertainty about underlying model may be due to over-branding or unclear labelling. Given that participants were unable to identify their model, this could suggest that factors other than validation status (which requires model identification) such as price, vendor recommendations, attractiveness of packaging or additional features (e.g. mobile application interconnectivity) are more important to patients when choosing a monitor. This further reiterates the need for improved education on choosing validated HBPMs for patients both by healthcare professionals and medical device vendors.

### Strengths and weaknesses of the study

A major strength of this study is that it describes the HBPM choices of a large group of patients with hypertension across the UK. Having a free-text entry box with a character limit hindered the identification of a significant number of HBPMs which otherwise would have added to the strength of our observations. This study has an inherent selection bias as it only included UK participants enroled in a long-term internet-only clinical trial. Like most randomised controlled trial cohorts, these participants represent a population who may be more engaged in their own healthcare [32]. Additionally, the online nature of the TIME study and higher recruitment from less deprived regions, is likely to have resulted in under-representation of people from lower socioeconomic groups. In this study we did not investigate the age or calibration status of HBPMs used by participants; both of these factors have been reported to affect the accuracy of BP measurements even in validated devices [33]. Prices of older HBPMs are likely to have been higher at the time of original purchase.

At the time of analysis, dabl and BIHS were the only publicly accessible databases of HBPMs validation status. Since 2019, STRIDE BP, an international scientific non-profit organisation, has listed validated HBPMs on its website, www.stridebp.org [34]. The continued transparent clinical and scientific oversight of the STRIDE BP and BIHS HBPM listings makes them most suitable for this type of research in future.

### Future research

Participants in the TIME study who reported owning their own HBPM were invited to submit home blood pressure measurements obtained using their HBPM through the TIME study web interface at intervals throughout the study. These measurements could be analysed to look for any evidence of variation in systolic and diastolic pressures associated with validation status. A qualitative study exploring the decision-making process of patients with hypertension when purchasing a HBPM may be warranted to determine factors which could influence the choice of HBPM. In addition, new HBPMs are becoming available, some with additional capabilities, such as detection of atrial fibrillation, or night-time blood pressures and it would be interesting to re-evaluate HBPM ownership again in the future.

## Conclusion

From this study, it is evident that patients with hypertension in the UK are using a large variety of HBPMs. Although most participants in the TIME study are using validated HBPMs, a significant number are using HBPMs which do not have clear evidence of validation. Amongst participants whose model of HBPM was identified, upper arm-type HBPMs were more common than wrist-type HBPMs with almost all participants using automated monitors. HBPMs with validation were more expensive than HBPMs without evidence of validation. However, socioeconomic status did not appear to affect likelihood of choosing a validated monitor. The factors which influence a patient’s choice of HBPM remain unclear, but the public may benefit from clearer labelling and information about validation status of HBPM to allow them to make more informed purchases.

### Summary table

#### What is known about topic

- Self-monitoring with home blood pressure monitors with guidance is associated with improved blood pressure control.
- Various HBPMs are available for purchase but only some are validated against independent standards.

#### What this study adds

- Patients with hypertension in the UK are using a large variety of home blood pressure monitors.
- A significant number of hypertension patients in the UK are using home blood pressure monitors lacking clear evidence of validation.
- Home blood pressure monitors with evidence of validation had a significantly higher median price compared to monitors lacking clear evidence of validation.
